# Overexpression of SOX4 correlates with poor prognosis of acute myeloid leukemia and is leukemogenic in zebrafish

**DOI:** 10.1038/bcj.2017.74

**Published:** 2017-08-25

**Authors:** J-W Lu, M-S Hsieh, H-A Hou, C-Y Chen, H-F Tien, L-I Lin

**Affiliations:** 1 Department of Clinical Laboratory Sciences and Medical Biotechnology, National Taiwan University, Taipei, Taiwan; 2 Division of Hematology, Department of Internal Medicine, National Taiwan University, Taipei, Taiwan; 3 Laboratory Medicine, National Taiwan University Hospital, Taipei, Taiwan

## Abstract

The SOX4 transcription factor is a key regulator of embryonic development, cell-fate decision, cellular differentiation and oncogenesis. Abnormal expression of SOX4 is related to malignant tumor transformation and cancer metastasis. However, no reports are available regarding the clinical significance of SOX4 in acute myeloid leukemia (AML) and the role of SOX4 in leukemogenesis. In the current study, we found that AML patients with low bone marrow (BM) SOX4 expression had higher remission rates and longer overall survival than those with high SOX4 expression, regardless of age, white blood cell count at diagnosis, karyotype profile and N*PM1*/*FLT3-*ITD status. To elucidate the role of SOX4 in leukemogenesis, we generated a transgenic zebrafish model that overexpressed human *SOX4* in the myeloid lineage Tg(*spi1*-*SOX4*-*EGFP*). These transgenic zebrafish showed, at 5 months of age, increased myelopoiesis with dedifferentiation in kidney marrow. At 9 months of age, their kidney structure was significantly effaced and distorted by increased infiltration of myeloid progenitor cells. These results suggest that SOX4 is not only an independent prognostic factor of AML, but also an important molecular factor in leukemogenesis.

## Introduction

The SOX4 belongs to the SOX (Sry-related high-mobility group box) family and has been characterized as a transcription factor.^1, 2^ Over the past decade, multiple functions of SOX4 have been unveiled, and the protein is now known to play important roles in embryonic development, cell-fate decision and cellular differentiation.^3, 4, 5, 6^ Overexpression and amplification of *SOX4* have been implicated in various cancers and are correlated with poor prognosis.^7, 8, 9, 10, 11, 12, 13, 14, 15, 16^ In mouse models, previous studies demonstrated that the upregulation of *Sox4* can be induced by and then cooperate with the aberrant expression of *AML1-ETO*,^17^ *NUP98-DDX10*^(ref. 18)^ and *PML-RARa*;^19^ the overexpression of *HOXA9*^(ref. 20)^ *CREB*^21^ and *Evi1*;^22^ and the haplosufficiency of *PU.1*^(ref. 23)^ to trigger leukemogenesis. Conversely, mice lacking *Sox4* have shown pro-B-lymphocyte expansion and defects in cardiac outflow tract formation.^24^ Furthermore, a previous study that employed retroviral transduction of *Sox4* and bone marrow transplantation techniques revealed that increased *Sox4* expression may cooperate with the deregulation of *Mef2c* expression to induce myeloid leukemia in recipient mice.^25^

Recently, *Sox4* gene was reported to be a direct target of C/EBPα (CCAAT/enhancer-binding protein-α). C/EBPα is known to inhibit the self-renewal of leukemic cells and to restore cellular differentiation. The overexpression of *Sox4* that results from C/EBPα inactivation contributes to the development of a type of leukemia that is characterized by a distinct leukemia-initiating cell phenotype. This recent work further indicated that *Sox4* is a key oncogenic target and critical mediator of C/EBPα mutants in acute myeloid leukemia (AML), suggesting a potential novel therapeutic approach to the treatment of this disease.^26, 27^ However, the clinical implications of *SOX4* expression and its role of AML leukemogenesis are not well understood.

The current study investigated the relationship between bone marrow (BM) SOX4 expression and clinicopathological parameters of *de novo* AML and also evaluated the prognostic value of SOX4 expression for AML patients. This is the first study to report on the prognostic implications of SOX4 expression for AML patients. Our immunohistochemical staining results illustrated that high BM SOX4 protein content is an independent unfavorable prognostic factor for overall survival. A meta-analysis that we conducted using an online data cohort retrieved from PrognoScan (a new database for meta-analysis of the prognostic value of genes; http://www.abren.net/PrognoScan/) revealed similar findings. Finally, as several zebrafish models have been proposed for study of hematopoiesis and myeloid malignancies in recent years,^28, 29, 30, 31, 32^ we used a transgenic *SOX4* zebrafish line to demonstrate, for the first time, that *SOX4* overexpression driven by *spi-1* (a myeloid-specific promoter) can lead to expanded myelopoiesis with leukemic phenotype.

## Materials and methods

### Study patients

From March 2009 to December 2011, a total number of 112 adult patients were enrolled in this study. This study was approved by the institutional review board of the National Taiwan University Hospital and written informed consent was obtained from all participants in accordance with the Declaration of Helsinki. See Supplementary Materials and Methods for details.

### Immunocytochemical staining of SOX4 protein

To assess SOX4 expression in leukemic cells, immunocytochemical staining was performed.^33^ See Supplementary Materials and Methods for details.

### Generation and husbandry of transgenic zebrafish

Zebrafish (*Danio rerio*) embryos, larvae and adult fish were maintained at 28 °C under continuous flow and a 14 h light/10 h dark cycle.^31^ All experiments involving zebrafish were approved by the institutional animal care and use committee of the National Taiwan University. The myeloid-specific *spi-1*-driven *SOX4* transgenic fish was generated as previously described.^29, 34^ The primer sequences are listed in Supplementary Table 1. See Supplementary Materials and Methods for details.

### Isolation of RNA as well as reverse transcription-PCR

Total RNA from various tissues or a total number of 30 embryos was isolated using NucleoSpin and TRIzol (Invitrogen, Carlsbad, CA, USA), then reverse transcribed into complementary DNA (cDNA) with the High-Capacity RNA-to-cDNA Kit (Applied Biosystems, Carlsbad, CA, USA) and finally amplified by PCR with KOD-FX Taq polymerase (Toyobo, Osaka, Japan), in accordance with the manufacturer’s instructions. See Supplementary Materials and Methods for details.

### Quantitative reverse transcription-PCR

Quantitative reverse transcription-PCR was carried out in an ABI 7500 Fast Real-Time PCR system (Applied Biosystems, Foster City, CA, USA) using SYBR green as the detection dye (Power SYBR Green PCR Master Mix, Applied Biosystems). PCR conditions and subsequently cycle thresholds (Ct) calculations were according to standard protocols. The primer sequences of various target genes are listed in Supplementary Table 1. See Supplementary Materials and Methods for details.

### Whole-mount *in situ* hybridization and whole mount immunohistochemical staining

Whole-mount *in situ* hybridization analysis was performed essentially as previously described^32^ using the antisense digoxigenin-labeled *myeloperoxidase* (*mpo*) RNA probe that was generated from the partial cDNA (901 bp) sequence of *mpo* was amplified by PCR with T3-*mpo*-F and T7-*mpo*-R primers (Supplementary Table 1). See Supplementary Materials and Methods for details.

For whole-mount immunohistochemical staining analysis, embryos were fixed, dehydrated and rehydrated as procedures for whole-mount *in situ* hybridization. Embryos were then incubated in the blocking solution, reacted with rabbit anti-GFP antibodies (1:100 dilutions; GTX113617, GeneTex, San Antonio, TX, USA), and then finally reacted with Alexa Fluor 488-labeled goat-anti rabbit secondary antibodies (1:100 dilutions; A-11008, Invitrogen). See Supplementary Materials and Methods for details.

### Tissue collection and histochemical analysis

Tissue collection and histochemical analysis were performed as previously described.^29^ See Supplementary Materials and Methods for details.

### Cytological analysis of kidney marrow and peripheral blood

Collection and cytological analysis of blood cells from kidney marrow (KM) and peripheral blood were performed as previously decribed.^29^ See Supplementary Materials and Methods for details.

### Flow cytometric analysis

Collection and flow cytometric analysis of blood cells from KM and peripheral blood were performed as previously decribed.^29^ See Supplementary Materials and Methods for details.

### Sudan black staining

Sudan black staining was performed as previously described.^35^ See Supplementary Materials and Methods for details.

### Myeloperoxidase staining

KM smears were fixed with pH 6.6 formalin-acetone (Muto Pure Chemicals Co., Ltd, Tokyo, Japan) for 30 s, washed with double-distilled water and then dried. Slides were subsequently treated with 3,3’-diaminobenzidine-tetrahydrochloride (Sigma Chem. Co., St Louis, MO, USA) for 15 min at room temperature. After being washed and dried, slides were counterstained with hematoxylin for 3 min.

### Statistical analysis

All statistical analyses performed for this study involved comparison between experimental and control groups using two-tailed Student’s *t*-tests, Mann–Whitney *U*-tests, one-way analysis of variance, χ^2^ test or Fisher’s exact test and multivariate analysis with Cox proportional hazards regression models as previously described.^36^ We used Kaplan–Meier estimation techniques to plot survival curves and log-rank tests to examine difference between groups as previously described.^37^ *P-*values of <0.05 were considered statistically significant. See Supplementary Materials and Methods for details.

## Results

### BM SOX4 expression as an independent prognostic factor of AML

**Figure 1:**
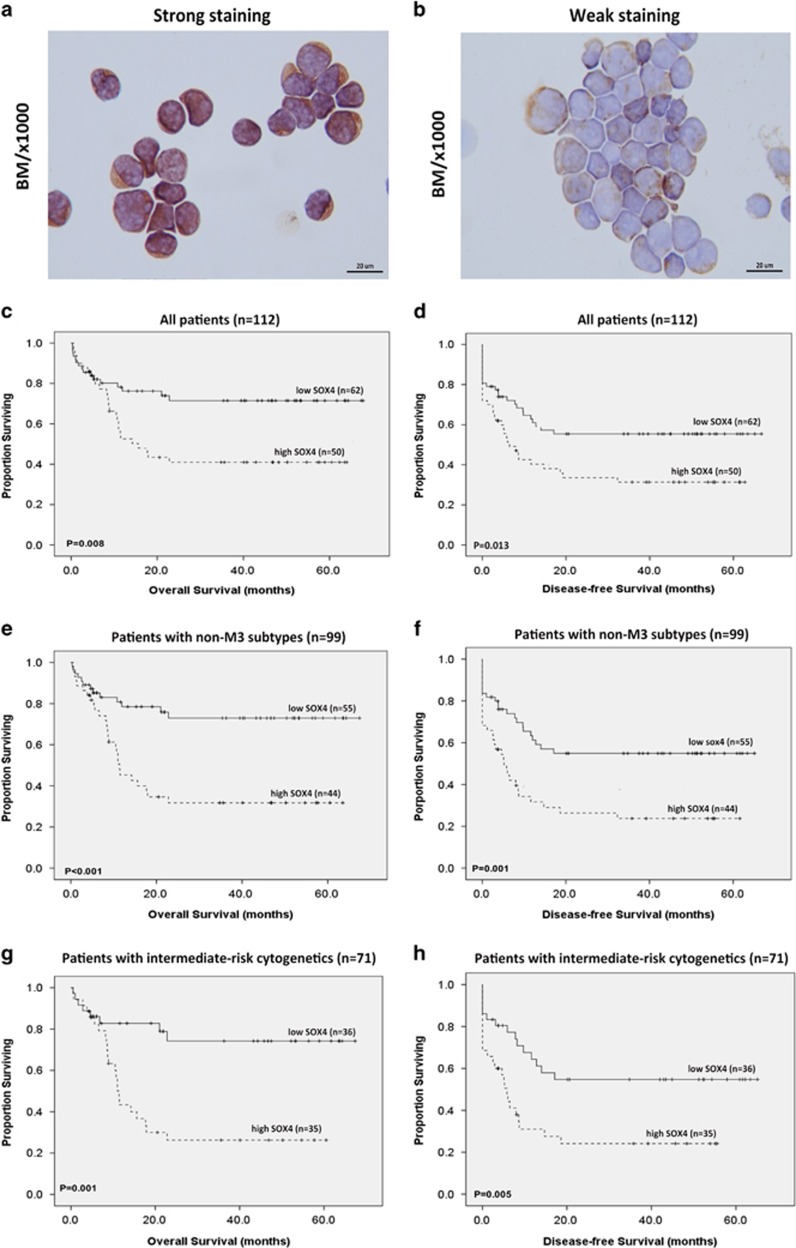

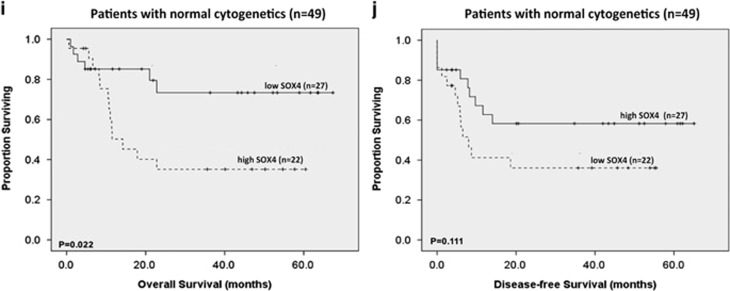
Kaplan–Meier survival analysis according to SOX4 expression in AML patients. Representative immunocytochemical stain of SOX4 protein in bone marrow specimens from patients with low SOX4 protein (**a**) and with high SOX4 protein (**b**). Overall survival and disease-free survival were analyzed in all patients with *de novo* AML (**c**, **d**), in patients with non-M3 subtypes (**e**, **f**), patients with intermediate-risk cytogenetics (**g**, **h**) and patients with normal cytogenetics (**i**, **j**). Number of patients and median survival time are indicated in each subgroup.

Results of immunocytochemical staining revealed that BM SOX4 expression varied greatly in AML patients. We divided patients into two groups according to the intensity and extent of SOX4 expression as follows: low expression group (score 0–2, *n*=62, Figure 1a) and high expression group (score 3–4, *n*=50, Figure 1b), respectively. The various clinical manifestations of AML did not show significant differences in terms of SOX4 expression (Supplementary Table 2); except that high SOX4 expression was somehow correlated with CD11b expression in leukemia cells (Supplementary Table 3), and AML patients with low SOX4 expression tended to have favorable-risk karyotyping (*P*=0.0866, Supplementary Table 2). We did not observe significant differences between the high and low expression groups in terms of age, gender, hemograms, *nucleophosmin-1* (*NPM1*) mutation and *FLT3*-ITD (internal tandem duplication of the *fms-like tyrosine kinase-3*). In addition, of the 112 AML patients who underwent conventional intensive induction chemotherapy, 85 (75.9%) achieved complete remission, and the high and low expression groups showed similar probabilities of achieving first complete remission (36/50, 72 vs 49/62, 79%, *P*=0.3219). However, high SOX4 expression was associated with increased relapse rates compared with low SOX4 expression (19/36, 52.8 vs 13/49, 26.5%, *P*=0.028).

Furthermore, with a median follow-up period of 46.7 months (range: 0.3–70.9 months), SOX4 expression was associated with overall survival and disease-free survival in all patients with *de novo* AML (*P*=0.008 and *P*=0.013, respectively, Figure 1c), patients with non-M3 subtypes (*P*<0.001 and *P*=0.001, respectively, Figure 1d), patients with intermediate-risk cytogenetics (*P*=0.001 and *P*=0.005 respectively, Figure 1e) or even in those with normal karyotype profile (*P*=0.022 and *P*=0.111, Figure 1f). In multivariate analysis, high SOX4 expression was found to be an independent poor prognostic factor of overall survival (relative risk 1.924, 95% confidence interval 1.020–3.628, *P*=0.043) irrespective of age, white blood cell count at diagnosis, karyotype profile and *NPM1*/*FLT3*-ITD status (Table 1).

### Generation of Tg(*spi1*:*SOX4*-*EGFP*) zebrafish lines by tol2 transposon system

**Figure 2:**
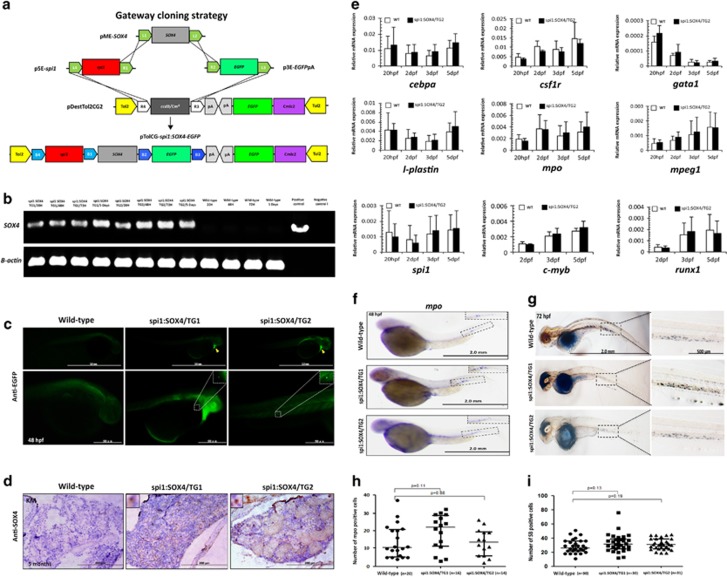
Generation and characterization of the Tg(*spi1:SOX4-EGFP*) transgenic zebrafish. (**a**) Schematic diagram showing the LR recombination reaction used to generate the expression constructs, including three entry clones (p5E-*spi1*, pME-*SOX4* and p3E-*EGFP*pA) and a destination vector (pDestTol2CG2) that contains the *cmlc2:EGFP*-pA expression cassette. The final construct (pTolCG-*spi1:SOX4-EGFP*) is shown at the bottom of the figure. (**b**) Results of semiquantitative PCR showing the expression of *SOX4* in TGs and wild-type embryos at 20, 48 and 72 h post fertilization (HPF) and at 5 days. Negative control: non-template; Positive control: pTolCG-*spi1:SOX4-EGFP* plasmid. (**c**) Whole-mount immunohistochemical staining (IHC) showed EGFP-positive cells in the heart (yellow arrow) as well as around the yolk sac and caudal hematopoietic tissue (CHT) at 48 HPF. The panel shows GFP+ cells at a higher magnification. (**d**) Tissue sections showing SOX4-positive cells in the KM of Tg(*spi1:SOX4-EGFP*) fish but not in those of wild-type fish. The panel shows SOX4-positive cells at a higher magnification. (**e**) Results from quantitative reverse transcription-PCR (RT-PCR) analysis of hematopoietic marker genes in *SOX4* transgenic zebrafish embryos and in wild-type fish embryos. Data are presented as the mean±s.e.m. from three independent experiments. (**f**, **g**) Results from whole-mount *in situ* hybridization (WISH) of *mpo* and Sudan black (SB) staining showing *mpo*-positive cells in CHT at 48 HPF and SB-positive cells in CHT at 72 HPF, respectively (magnification: × 40). The panel shows positive cells at a higher magnification (× 100), respectively. Quantification of *mpo*-positive cells (**h**) and SB-positive cells (**i**). Differences among variables were assessed using Student’s *t*-test.

To further investigate the role of SOX4 in leukemogenesis, we use Multisite Gateway (Invitrogen, Grand Island, NY, USA) and Tol2 transposon methods to generate pTolCG-*spi1*:*SOX4*-*EGFP* constructs. In these constructs, *SOX4* and *EGFP* expression is controlled by the myeloid-specific *spi1* (also known as *pu.1*) promoter. The pTolCG-*spi1*:*SOX4*-*EGFP* constructs were then coinjected with Tol2 transposase mRNA into one-cell embryos of wild-type AB strain zebrafish to generate transgenic zebrafish founder, namely Tg(*spi1:SOX4*-*EGFP*) (Figure 2a). Two transgenic *SOX4* lines (TG1 and TG2) were generated in parallel. F1 fish were generated by crossing *SOX4* founders and wild-type zebrafish, and *SOX4* transgene expression was demonstrated by semi-quantitative PCR (Figure 2b). The expression of *SOX4-EGFP* fusion proteins was verified using whole-mount immunohistochemical staining with anti-EGFP antibodies. In contrast to wild-type fish, which showed no enhanced green fluorescent protein (EGFP), the Tg(*spi1:SOX4-EGFP*) fish exhibited EGFP in the yolk sac and in caudal hematopoietic tissue after 48 h post fertilization (Figure 2c). We further investigated SOX4 expression by applying immunohistochemistry staining anti-SOX4 on tissue sections of 5-month-old Tg(*spi1:SOX4*-*EGFP*), and found that SOX4 expression was restricted to a small fraction of cells in the KM (Figure 2d).

### Tg(*spi1:SOX4*-*EGFP*) zebrafish embryos underwent normal hematopoiesis

To assess whether *SOX4* overexpression driven by *spi1* has an effect on early hematopoietic processes in transgenic zebrafish, several primitive and definitive markers including *cebpa*, *csf1r*, *gata1*, *l-plastin*, *mpo*, *mpeg1*, *spi1*, *c-myb* and *runx1* were examined by quantitative reverse transcription-PCR analysis. We found that there were no significant differences of these hematopoiesis-related transcription factors in transgenic zebrafish at both primitive and definitive stages (Figure 2e). Further whole-mount *in situ* hybridization experiments revealed that comparable numbers of *mpo*-positive cells were located in the aorta-gonad-mesonephros and caudal hematopoietic tissue regions in both *SOX4* and wild-type fish at 48 h post fertilization (Figures 2f and h). Sudan black staining reveled similar results for granulocyte colonized in caudal hematopoietic tissue at 72 h post fertilization (Figures 2g and i). These results indicate that *SOX4* transgenic zebrafish were undergoing normal hematopoietic processes during the larval stage.

### Myeloid-specific expression of *SOX4* results in expanded myelopoiesis with poor differentiation in adult zebrafish

Myeloid malignancies are known to result from multiple long-term processes. Therefore, long-term examination of adult zebrafish was reasonable even though the overexpression of *SOX4* did not influence early hematopoiesis. In this study, biopsy sections of kidney tissues showed mild vacuoles in renal tubule structure in 5-month-old *SOX4* fish; moreover, kidneys showed mild or moderate effacement, a distorted structure and increased infiltration of myeloid cells in 9- and 12-month-old *SOX4* fish (Supplementary Figure 1).

**Figure 3:**
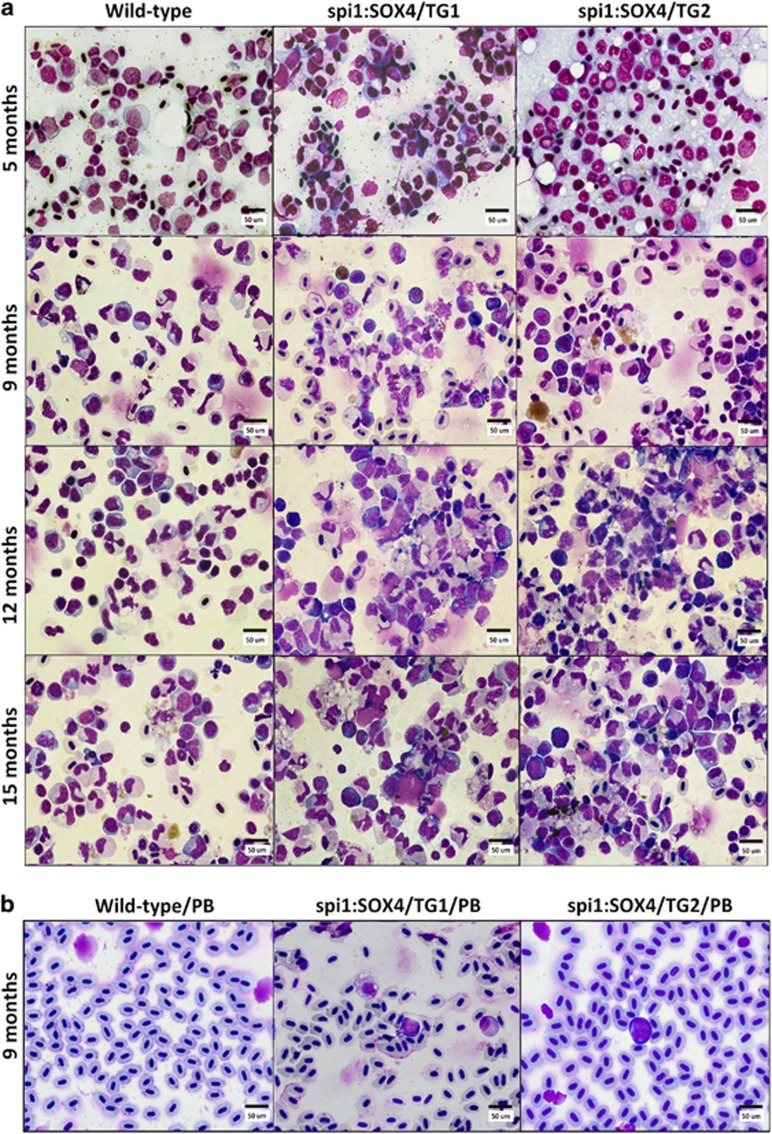
Morphological analysis of various blood cell types harvested from the KM and peripheral blood of Tg(*spi1:SOX4-EGFP*) zebrafish at indicated age. Tg(*spi1:SOX4-EGFP*) KM smears showed myeloid hyperplasia with increased production of myeloblasts in an age-dependent manner (**a**) and their peripheral blood smears showed immature myeloid cells (**b**). Conversely, wild-type fish showed normal hematopoiesis with adequate maturation (magnification: × 1000).

Microscopic observation of KM smears from Tg(*spi1:SOX4*-*EGFP*) fish were analyzed at the ages of 5, 9, 12 and 15 months. The KM of 5-month-old *SOX4* zebrafish was comparable with the KM of age-matched 5-month-old AB wild-type fish; however, KM of *SOX4* transgenic fish demonstrated a greater number of myeloid progenitors and an excess of blast cells with focal aggregation at 9, 12 and 15 months, implying that myeloid transformation is age dependent (Figure 3a). These blast cells in KM were generally of medium to large size and were characterized by a mildly basophilic cytoplasm with scanty or fewer granule as well as a high nucleus-to-cytoplasm ratio (Figure 3a). In addition, a few typical blast cells in peripheral blood were observed in 9-month-old *SOX4* fish (Figure 3b).

**Figure 4:**
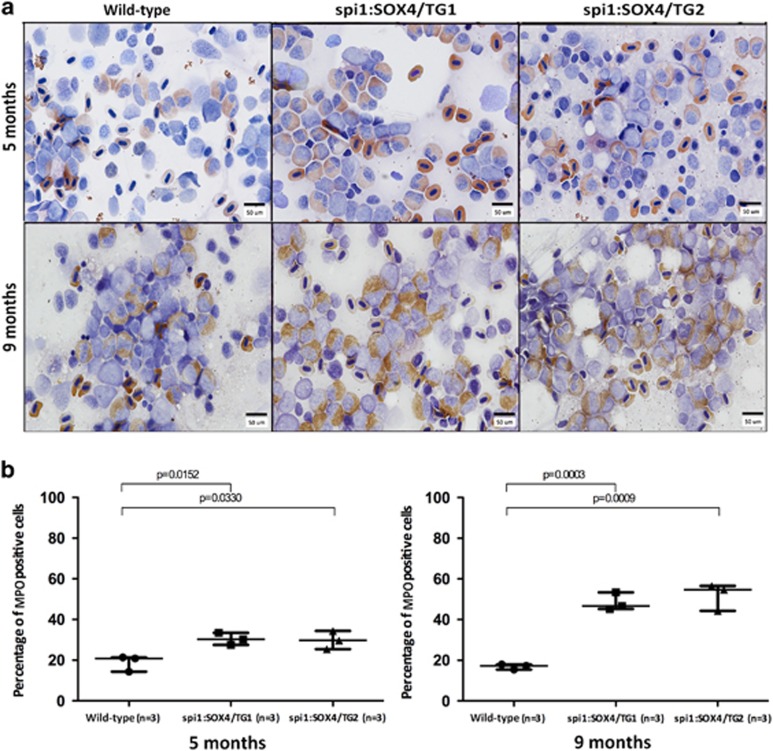
Myeloperoxidase (MPO) staining analysis of *spi1:SOX4-EGFP* zebrafish. (**a**) Increased myeloperoxidase was detected in *SOX4* transgenic fish at 5 and 9 months (magnification: × 1000). (**b**) Quantification of MPO-positive cells. Differences among variables were assessed using Student’s *t*-test.

On microscopic examination of blood cells in KM smears, we found that compared with age-matched 9-month-old AB wild-type fish, the KM of 9-month-old *SOX4* zebrafish showed a greater myeloid-to-erythroid ratio, a higher number of myeloid progenitors and decreased number of both immature and mature erythroid cells. Additional significant differences in myeloid-to-erythroid ratios and in all blood components of KM were found in 12- and 15-month-old transgenic *SOX4* zebrafish (Table 2). Similar results were demonstrated using an additional flow cytometric analysis (Supplementary Figure 2). In addition, MPO staining further confirmed that there were significantly more myeloid cells in *SOX4* fish compared with wild-type control fish of the same age (Figure 4). Taken together, our findings reveal that continuous overexpression of *SOX4* led to an expansion of myeloid cells in the KM of adult fish, and that the majority of accumulated myeloid cells were immature.

## Discussion

To the best of our knowledge, this is the first study to report that increased BM SOX4 expression is an independent poor prognostic factor of overall survival in AML patients, irrespective of age, white blood cell count, karyotype profile and other genetic markers. The poor prognosis associated with high BM SOX4 expression was also demonstrated in CN-AML patients. These results are consistent with an online transcriptome data (GSE12417-GPL96 cohort) from AML patients (Supplementary Figure 3). Their data were analyzed by PrognoScan, a new database in which meta-analyses to elucidate the prognostic value of genes can be performed. It demonstrated that higher *SOX4* mRNA expression led to a worse overall survival in AML patients (hazard ratio 1.21, 95% confidence interval 1.01–1.47, *P*=0.043). Zhang *et al.*^26^ previously reported that SOX4 is a direct target and crucial mediator of C/EBPα mutants in AML. It is therefore possible that poor prognosis is associated with *CEBPA* mutants or *CEBPA* methylation. However, in our previous report, we found that AML patients with *CEBPA* double mutations^38^ or higher *CEBPA* methylation^39^ had a superior response to chemotherapy treatment, and similar findings were reported in studies by other researchers as well.^40, 41, 42^ The different prognoses associated with SOX4 expression and *CEBPA* gene status may be explained by the fact that *SOX4* expression is also associated with other genetic abnormities including *MOZ-TIF2*, *AML1-ETO*, *NUP-HOXA9* and *FLT3-*ITD.^26, 43^ Of these abnormalities, *AML1-ETO* was found to be a better prognostic factor for AML,^44, 45^ and *FLT3*-ITD was found to be a poor prognostic factor.^46^ Therefore, the conflicting results could result from the complexity, heterogeneity and multifactorial nature of AML. In addition, results from a cancer microarray database and integrated data (Oncomine; https://www.oncomine.org/resource/login.html) showed that leukemia cells of AML patients (*n*=542) expressed significantly more *SOX4* than did normal cells from controls (*n*=74) (GSE13164 cohort; Supplementary Figure 4a). Significant differences between M1 and other FAB (French–American–British) categories (GSE14468 cohort) were also found (Supplementary Figure 4b). The mechanisms underlying the increase in SOX4 expression independently associated with poor prognosis and various genetic aberrations still require further study. Nonetheless, complementing chemotherapy with agents that target SOX4 could be beneficial treatment strategy for AML patients.

Zebrafish sox4 proteins (sox4a: accession number. NP_957195.1 and sox4b: accession number NP_998287.1) share a high level of amino acid similarity with human SOX4 (accession number: NP_003098.1) and mouse Sox4 (accession number: NP_033264.2), particularly within the HMG box (sox4a: 100%, sox4b: 97.4%) and the TAD/DD (sox4a: 91.2%, sox4b: 70.6%) functional domains (Supplementary Figure 4a). Phylogenetic analysis revealed that the zebrafish sox4a/b is homologous with SOX4 proteins from other species (Supplementary Figure 5b). The high conservation of SOX4 among humans, mice and zebrafish suggests that SOX4 proteins could share similar functions. We also characterized the mRNA expression of *sox4a* and *sox4b* in early developmental stages of transgenic zebrafish. In so doing, we found that the expression levels of *sox4b* and *sox4a* in transgenic zebrafish were comparable to those of wild-type fish (data not shown).

The kidneys of Tg(*spi1:SOX4-EGFP*) zebrafish began showing mild vacuoles in renal tubule structure at the age of the 5 months. At 9 and 12 months, the kidneys further showed mild or moderate effacement, a distorted structure and increased infiltration of myeloid cells. Microscopic examination revealed that the KM blood cells of 5-month-old Tg(*spi1*:*SOX4*-*EGFP*) zebrafish were comparable with those of age-matched AB wild-type fish. However, at 9, 12 and 15 months, KM cells from Tg(*spi1*:*SOX4*-*EGFP*) fish had a greater number of myeloid progenitors and an excess of blast cells with focal aggregation than did KM cells of age-matched AB wild-type fish, implying that myeloid transformation was age dependent. This is the first study to report on transgenic *SOX4* zebrafish with myeloid transformation phenotype.

Results from previous research in mouse model studies support our findings. For example, in one study, 73% of mice developed myeloid leukemia after receiving a transplant of *MSCV-Sox4*-infected marrow, whereas none of the control mice developed leukemia.^25^ Other reports have also found that *Sox4* leads to myeloid malignancies in mouse model as follows. *Sox4* may upregulate *CREB* expression and cooperate with *CREB* to activate downstream *cyclin D1*, *bcl-2* and *c-fos* that in turn increases cell proliferation.^21^ *Sox4* may also downregulate *Pu.1*, leading to *Pu.1* haploinsufficiency, which accelerates and increases the penetrance of *Sox4*-induced leukemia.^23^ A later study revealed that a novel PAD4/SOX4/PU.1 signaling pathway is involved in the differentiation of leukemic cells into granulocytes.^47^ Furthermore, in an AKXD23 mouse model of myeloid leukemia, proviral insertions to *Sox4* and *Evi1* were found to cause increases in *Sox4* and *Evi1* expressions, respectively; and *Sox4* acted alone or cooperated with *Evi1* to induce transcriptional activation and subsequent cell proliferation.^22^ Findings from that research also demonstrated that overexpression of *Sox4* in 32Dcl3 cells markedly inhibited cytokine-induced granulocyte maturation and associated cell proliferation.^22^ Finally, Fung *et al.*^27^ suggested that activation of the Wnt pathway, activation of the TGF-FOXO pathway and epigenetic aberration of *Sox4* could be responsible for myeloid leukemogenesis.

In conclusion, we reveal that BM SOX4 expression could serve as an informative new biomarker for the clinical prognosis of AML patients. Moreover, we demonstrate that in zebrafish, the myeloid-specific expression of *SOX4* can induce leukemic phenotype. Therefore, we suggest that treating AML patients with agents that target SOX4 or its downstream molecules in addition to chemotherapy could be an effective therapeutic strategy. Furthermore, this zebrafish disease model could be useful for studying gene functions involved in myeloid malignancies and to be a valuable *in vivo* platform for screening antileukemic drugs.

## Figures and Tables

**Table 1:** Multivariate analysis (Cox regression) of SOX4 expression on the overall survival and disease-free survival

*Variables*	*Overall survival*				*Disease-free survival*			
		*95% CI*				*95% CI*		
	*RR*	*Lower*	*Upper*	P *value*	*RR*	*Lower*	*Upper*	P *value*
Age^a^	1.023	1.002	1.044	0.034	1.012	0.994	1.029	0.203
WBC^b^	1.004	1.000	1.007	0.029	1.004	1.001	1.006	0.012
Karyotype^c^	3.038	1.188	7.771	0.020	2.127	1.105	4.096	0.024
*NPM1/FLT3-*ITD^d^	1.197	0.510	2.811	0.680	1.345	0.651	2.778	0.423
High SOX4^e^	1.924	1.020	3.628	0.043	1.663	0.983	2.813	0.058

Abbreviations: CI, confidence interval; RR, relative risk; WBC, white blood cell.

a The risk by 1 year older in age.

b The risk by 1000/μl increase in WBC.

c Unfavorable cytogenetics vs others.

d *NPM1*^mut^/*FLT3-*ITD^neg^ vs other subtypes.

e High SOX4 expression vs low SOX4 expression.

**Table 2:** Morphological analysis of blood cell types in the kidney marrow from *SOX4* transgenic fish compared with wild type

*Total number of zebrafish*^a^ (n=*65)*	*Myeloid progenitors (blasts/blast-like; %)*	*Myelomonocyte/neutrophil (%)*	*Lymphocyte (%)*	*Immature erythroid (%)*	*Mature erythroid (%)*	*M/E ratio*
5M-wild-type (*n*=5)	5.27±0.74	45.00±5.36	15.33±1.36	10.27±1.82	24.13±3.19	5.08±1.57
9M-wild-type (*n*=5)	5.73±0.64	39.67±5.01	14.39±2.97	12.27±0.83	27.93±6.03	3.72:1±0.53
12M-wild-type (*n*=5)	7.00±1.10	42.07±3.18	16.20±1.12	10.06±2.55	24.67±4.16	4.61±0.64
15M-wild-type (*n*=6)	7.11±1.88	40.95±2.33	15.95±2.04	11.28±1.04	25.44±4.06	4.30±0.47
5M-*SOX4* (*n*=12)#	7.64±2.56	37.53±5.22	13.33±1.65*	11.95±3.02	29.08±6.40	4.10±1.59
9M-*SOX4* (*n*=12)#	14.86±7.07**	50.53±10.33*	9.69±1.65**	7.22±3.46*	17.78±5.19*	11.62±7.25**
12M-*SOX4* (*n*=11)#	16.24±7.25**	58.33±7.14**	6.09±3.61**	6.21±2.16*	13.12±4.38**	13.55±5.45**
15M-*SOX4* (*n*=9)#	14.19±5.16**	50.74±9.15*	9.59±1.38***	7.15±3.29*	16.74±4.42**	10.48±4.08**

Abbreviations: M, month; M/E ratio, myeloid-to-immature erythroid ratio.

Significant differences between the *SOX4* transgenic fish and wild-type are indicated (*P<0.05; **P<0.01; ***P<0.001) by Mann–Whitney *U*-test.

Significant difference of groups between the *SOX4* transgenic fish and wild-type are indicated (^#^*P*<0.001) by one-way analysis of variance (ANOVA).

a A total number of 300 cells per zebrafish was counted to classify the distribution and subtypes of these hematopoietic cells.
