# Supplementary material for: Overexpression of SOX4 correlates with poor prognosis of acute myeloid leukemia and is leukemogenic in zebrafish
Source: Blood Cancer J. 2017 Aug 25;7(8):e593–. doi: 10.1038/bcj.2017.74 (PMC5596385; doi:10.1038/bcj.2017.74)
Supplement: Supplementary Materials [file bcj201774x1.pdf]

Supplementary materials

Material and Methods

Study Patients

From Mar 2009 to Dec 2011, a total number of 112 adult patients were enrolled in this study. Enrolled patients met the following criteria: they were newly diagnosed with *de novo* AML, they received standard treatment protocol, and they had enough cryopreserved cells for analysis at the National Taiwan University Hospital (NTUH). Among these patients, 98 received standard induction chemotherapy (Idarubicin 12 mg/m^2 per day on days 1-3 and Cytarabine 100 mg/m^2 per day on days 1-7) followed by consolidation chemotherapy with 2-4 courses of high-dose Cytarabine (2000 mg/m^2 q12h on days 1-4, with a total of 8 doses overall), with or without an anthracycline (Idarubicin or Novatrone) after achieving complete remission (CR). Patients with acute promyelocytic leukemia (M3 subtype) received concurrent all-trans retinoic acid and chemotherapy. This study was approved by the Institutional Review Board (IRB) of the NTUH and written informed consent was obtained from all participants in accordance with the Declaration of Helsinki.

Immunocytochemical staining of SOX4 protein

To assess SOX4 expression in leukemic cells, immunocytochemical staining was performed[1]. For this, cytospin smears of BM leukemia cells from the 112 patients were fixed for 3 min in formalin acetone (3%). The specimens were then incubated with peroxidase blocking enzyme (10 min), followed by rabbit polyclonal antibodies against human SOX4 overnight at 4°C (1:100 dilution; GTX82513, GeneTex, San Antonio, TX). Biotinylated donkey anti-rabbit IgG (1 g/ml, diluted in blocking solution,

30 min; DAKO, Carpinteria, CA) was used as the secondary antibody, and proteins were detected using the streptavidin-peroxidase complex (DAKO). Finally, the specimens were counter-stained with hematoxylin. An overall score of 0 to 4 was calculated for each specimen. This overall score was the sum of the staining intensity score (0: none, 1: weak, 2: strong) and a score that described the percentage of positively stained myeloid cells (0: ≤25%, 1: 26-50%, 2: >50%)

Generation and husbandry of transgenic zebrafish

Zebrafish (*Danio rerio*) embryos, larvae, and adult fish were maintained at 28°C under continuous flow and a 14-hour light/ 10-hour dark cycle[2]. All experiments involving zebrafish were approved by the Institutional Animal Care and Use Committee (IACUC) of the National Taiwan University (NTU). The transgenic founders were created using the Tol2 transposon system as described previously [3] and reared to sexual maturity. The Tol2 construct containing *spi-1*-controlled *SOX4* was generated as described previously [4]. The human *SOX4* gene was amplified by PCR (using cDNA from MV4-11 as a template) with the attB1-*SOX4*-F and attB2-*SOX4*-R primers but no stop codon. The primer sequences are listed in supplemental Table 1.

Isolation of RNA as well as reverse-transcription-PCR

Total RNA from various tissues or a total number of 30 embryos was isolated using NucleoSpin and TRIzol (Invitrogen, Carlsbad, CA). One microgram RNA was then reverse-transcribed into cDNA with the High Capacity RNA-to-cDNA Kit (Applied Biosystems, Carlsbad, CA). Following the reverse transcription reaction, the cDNA template was amplified by polymerase chain reaction (PCR) with KOD-FX Taq

polymerase (TOYOBO, Osaka, JAPAN), in accordance with the manufacturer's instructions. PCR products were then subjected to 2.0% agarose gel electrophoresis, and actin was used as an internal control for the cDNA assay.

Quantitative reverse-transcription polymerase chain reaction (qRT-PCR)

Quantitative reverse-transcription PCR (Q-RT-PCR) was carried out in an ABI 7500 Fast Real-Time PCR system (Applied Biosystems, Foster City, CA) using SYBR green as the detection dye (Power SYBR®Green PCR Master Mix, Applied Biosystems). PCR conditions consisted of 1 cycle at 50°C for 2 min and 95°C for 10 min, followed by up to 40 cycles at 95°C for 15 s (denaturation) and 60°C for 1 min (annealing/extension). Primer specificity was subsequently confirmed by dissociation curves. The primer sequences of various target genes are listed in Supplemental Table 1. The cycle thresholds (Ct) were determined, and their transcript levels were normalized according to *β-actin*. Specifically, normalization was calculated using the ΔC_T method, as follows: relative expression = $2^{-\Delta C_T}$, where $\Delta C_T = C_{T(target)} - C_{T(actin)}$.

Whole mount in situ hybridization (WISH) and immunohistochemical staining-whole mount (IHC-wm)

For WISH analysis, the partial cDNA (901 bp) sequence of *mpo* was amplified by PCR with T3-*mpo*-F and T7-*mpo*-R primers (supplemental Table 1). The antisense digoxigenin (DIG)-labeled *mpo* RNA probe used for WISH was generated by an *in vitro* transcription from this amplified partial *mpo* cDNA according to the manufacturer's instructions (DIG RNA Labeling Kit; Roche Applied Science). Embryos and larvae were fixed in 4% paraformaldehyde (PFA) and dehydrated. After stepwise rehydration, the

embryos were incubated in prehybridization buffer at 65°C, followed by overnight incubation with DIG-labeled *mpo* RNA probe at 65°C. The embryos were washed and incubated with alkaline phosphatases (AP)-conjugated anti-DIA antibody (Roche Applied Science) for overnight at 4°C. Blue color was developed using NBT/BCIP (Roche Applied Science) as substrate and the reaction was stopped with 0.5mM EDTA in PBST[5].

For IHC-wm analysis, embryos were fixed, dehydrated and rehydration as procedures for WISH. Embryos were then incubated in the blocking solution (10% normal goat serum in PBST) for 1 hour at room temperature, followed rabbit anti-GFP antibodies (1:100 dilutions; GTX113617, GeneTex, San Antonio, TX) overnight at 4°C. Following this, embryos were washed with PBS and incubated with Alexa Fluor 488-labeled goat-anti rabbit secondary antibodies for 2 hours at room temperature (1:100 dilutions; #A-11008, Invitrogen, Carlsbad, CA).

Tissue collection and histochemical analysis

Adult zebrafish at indicated stages were anesthetized by 0.02% tricaine (Sigma), and various organs were collected and fixed in 10% formalin overnight. The fixed tissues were then embedded in paraffin, sectioned into 5-μm thick sections, and mounted on poly-L-lysine coated slides. For immunohistochemistry, the sections on slides were incubated at 4°C overnight with primary rabbit anti-SOX4 antibodies (1:100 dilutions; GTX82513, GeneTex, San Antonio, TX). After washing with PBS, sections were developed using the EnVision™+ Dual Link System (Dako, Carpentaria, CA), and counterstained with hematoxylin before being dehydrated, cleared, and mounted with slide covers for examination under an Olympus BX51 microscope[4].

Cytological analysis of kidney marrow and peripheral blood

Control wildtype and transgenic fish were euthanized at 5, 9, 12 and 15 months of age. Peripheral blood (PB) was obtained by puncturing the tail using micropipette tips coated with heparin. Blood samples were immediately placed in a mixture of 0.9× PBS and 5% FBS. Cytospin smears of KM and PB were prepared for further cytological analysis. Liu's staining was then performed, and a total number of 300 cells per animal were examined under an Olympus BX51 microscope. Under microscopic observation, cells were identified as either myeloid progenitors, myelomonocytes, lymphocytes, immature erythrocytes or mature erythrocytes [4].

Flow cytometric analysis

Collection of PB and KM were performed as previously described[4]. Briefly, cell suspensions were subjected to LS-RII flow cytometry (BD Bioscience, San Jose, CA) at room temperature. For this, cell size and granularity were determined by forward scatter (FSC; abscissa) and side scatter (SSC; ordinate), respectively; and FlowJo (Tree Star) software was used for further analysis. Specifically, a total number of 30,000 cells per animal were analyzed and categorized into various subtypes of blood cells. Gated populations were as follows: immature and mature erythrocytes, lymphocytes, myelomonocytes, and precursor cells.

Sudan Black staining

Sudan black staining was performed as previously described[6]. Briefly, embryos or larvae were fixed for 2 hours in 4% paraformaldehyde (PFA) at room temperature,

washed in PBS, and stained with Sudan Black solution for 30 mins. Specimens were then washed twice (15 minutes per wash) with EtOH (70%). Following this, embryos were incubated in a mixture of 3% H_2O_2 and 1% KOH for 15 mins, and then rinsed by three times with PBST.

Myeloperoxidase staining

Kidney marrow smears were fixed with pH 6.6 formalin-acetone (Muto Pure Chemicals Co., Ltd.; Tokyo) for 30 secs, washed with ddH_2O, and then dried. Slides were subsequently treated with 3,3'-diaminobenzidine-tetrahydrochloride (Sigma Chem. Co., St Louis, MO) for 15 mins at room temperature. After being washed and dried, slides were counter-stained with hematoxylin for 3 mins.

Statistical analysis

All statistical analyses performed for this study involved comparison between experimental and control groups using two-tailed Student's t-tests, Mann-Whitney U tests, or one-way ANOVA. Discrete variables (i.e. high versus low SOX4 expression) were compared using the Chi-square test or Fisher's exact test. Disease-free status was defined as CR without relapse at the end of the study period, and disease-free survival (DFS) was defined as the interval of time that relapsed between recruitment to the first of three events: treatment failure, leukemia relapse, or death from any cause. To exclude confounding influences of different treatment regimens, patients who received allogeneic HSCT were censored on the day of cell infusion[7]. We used

Kaplan-Meier estimation techniques to plot survival curves and log-rank tests to examine difference between groups. Variables considered in multivariate analysis[8] included the following: including age, white blood cell (WBC) count at diagnosis, karyotype profile, *NPM1/FLT3*-ITD status and SOX4 expression. Relative risk (RR) values and 95% confidence intervals (CI) of independent risk factors associated with survival in multivariate analyses were estimated using Cox proportional hazards regression models. *P* values of less than 0.05 were considered statistically significant.

References

1. Hou HA, Chou WC, Lin LI, Tang JL, Tseng MH, Huang CF*, et al.* Expression of angiopoietins and vascular endothelial growth factors and their clinical significance in acute myeloid leukemia. *Leuk Res* 2008 Jun; **32**(6)**:** 904-912.

2. Lu JW, Hsieh MS, Liao HA, Yang YJ, Ho YJ, Lin LI. Zebrafish as a Model for the Study of Human Myeloid Malignancies. *Biomed Res Int* 2015; **2015:** 641475.

3. Kwan KM, Fujimoto E, Grabher C, Mangum BD, Hardy ME, Campbell DS*, et al.* The Tol2kit: a multisite gateway-based construction kit for Tol2 transposon transgenesis constructs. *Dev Dyn* 2007 Nov; **236**(11)**:** 3088-3099.

4. Lu JW, Hou HA, Hsieh MS, Tien HF, Lin LI. Overexpression of FLT3-ITD driven by spi-1 results in expanded myelopoiesis with leukemic phenotype in zebrafish. *Leukemia* 2016 Jun 7.

5. He BL, Shi X, Man CH, Ma AC, Ekker SC, Chow HC*, et al.* Functions of flt3 in zebrafish hematopoiesis and its relevance to human acute myeloid leukemia. *Blood* 2014 Apr 17; **123**(16)**:** 2518-2529.

6. Walters KB, Green JM, Surfus JC, Yoo SK, Huttenlocher A. Live imaging of neutrophil motility in a zebrafish model of WHIM syndrome. *Blood* 2010 Oct

14; **116**(15)**:** 2803-2811.

7. Hou HA, Lin CC, Chou WC, Liu CY, Chen CY, Tang JL*, et al.* Integration of cytogenetic and molecular alterations in risk stratification of 318 patients with de novo non-M3 acute myeloid leukemia. *Leukemia* 2014 Jan; **28**(1)**:** 50-58.

8. Tsai CH, Hou HA, Tang JL, Liu CY, Lin CC, Chou WC*, et al.* Genetic alterations and their clinical implications in older patients with acute myeloid leukemia. *Leukemia* 2016 Jul; **30**(7)**:** 1485-1492.

Supplementary Figure legends

Figure S 1. Histological analysis of the kidney of Tg(*spi1:SOX4-EGFP*) zebrafish. H&E stains of the kidney are from 5-, 9- and 12-month-old Tg(*spi1:SOX4-EGFP*) and wild-type fish. The kidney from *spi1:SOX4-EGFP* fish showed infiltration by myeloid cells at 9 and 12 -months. Arrow: renal tubule. Yellow triangle: infiltration cells. Magnification: 400x.

Figure S 2. FACS analysis and morphological analysis of hematopoietic cells from (A, C, E, G) the kidney marrow of Tg(*spi1:SOX4-EGFP*) and (B, D, F, H) wild-type zebrafish. A total number of 30,000 cells per animal were analyzed by flow cytometry to differentiate various subtypes of hematopoietic cells. Gate populations were as follows: erythroid, lymphocytes, myelomonocytes, and precursor cells. Mean percentage of cells is indicated for each gated subpopulation. FSC-A, forward scatter-area; SSC, side scatter-area.

Figure S 3. Kaplan–Meier overall survival curves generated from 163 patients in the AML cohort (GSE12417-GPL96) according to *SOX4* expression in PrognoScan (* indicates that $P<0.05$).

Figure S 4. *SOX4* mRNA expression in (A) normal PBMC and leukemic cells from patients with AML (dataset GSE13164) and (B) patients with various FAB subtypes (dataset GSE14468) in Oncomine (* indicates that $P<0.05$).

Figure S 5. Amino acid sequence alignment of human, mouse, and zebrafish SOX4 proteins. (A) Conservation scoring and (B) phylogenetic analysis were performed by PRALINE (http://www.ibi.vu.nl/programs/pralinewww/) and Phylogeny.fr (http://www.phylogeny.fr/), respectively.

Table. S1 Oligonucleotides primers used in this study

Gene Name	Primers name	Start	Sequence (5' to 3')	Accession number	Size (bp)
**Gateway recombination transgenesis**					
SOX4	attB1-*SOX4*-F	1	GGGGACAAGTTTGTACAAAAAAGCAGGCTATGGTGCAGCAAACCAACAA	NM_003107.2	1423
	attB2-*SOX4*-R	1423	GGGGACCACTTTGTACAAGAAAGCTGGGTAGTAGGTGAAAACCAGGTTGG		
**Primitive & definitive markers**					
mpo	Q-*mpo* -F	1668	CCGTGGATTGATTGGTCGTC	NM_212779.1	220
	Q-*mpo* -R	1887	CACCACAGCCAATTCTTGCT		
spi1	Q-*spi1*-F	865	GTTCCTGCTTGACCTTCTGCGAAA	XM_005168850.2	176
	Q-*spi1*-R	1440	TCAGTGCTCTTGCCATCTTCTGGT		
csf1r	Q-*csf1r -F*	113	TTCGCGCTCTTATTCCTCAT	NM_131672.1	200
	Q-*csf1r -R*	312	TTGGAAATGTAGCGCTTGTG		
mpeg1	Q-*mpeg1*-F	1345	ACTGCCATTCCTGTGGTTTC	NM_212737.1	199

	Q-*mpeg1*-R	1543	GGACAACTGCTGGATTTGGT		
cebpa	Q-*cebpa*-F	1080	AACGGAGCGAGCTTGACTT	NM_131885.2	250
	Q-*cebpa*-R	1329	AAATCATGCCCATTAGCTGC		
c-myb	Q-*c-myb*-F	1883	GCTGACTAGCTCTGTGCTGATG	NM_001309822.1	184
	Q-*c-myb*-R	2066	GCTGAGGTATTTGTGCGTGG		
runx1	Q-*runx1*-F	50	TTGGGACGCCAAATACGAACC	NM_131603.2	294
	Q-*runx1*-R	343	ATATCACCAAGGGCAACCACC		
l-plastin	Q-*l-plastin*-F	573	GCCCTTCACCATACAGGAGA	NM_131320.2	198
	Q-*l-plastin*-R	770	AGCAGAGCGATCAGAGCTTC		
gata1	Q-*gata1*-F	210	ATGAACCTTTCTACTCAAGCT	NM_131234.1	401
	Q-*gata1*-R	610	GCTGCTTCCACTTCCACTCAT		
**Internal control**					
B-actin	Q-*B-actin*-F	893	CTCCATCATGAAGTGCGACGT	NM_131031.1	180
	Q-*B-actin*-R	1072	CAGACGGAGTATTTGCGCTCA		

**WISH**					
mpo	T3-*mpo*-F		AATTAACCCTCACTAAAGGGAGATTTCTCAGCCAAAAGGATGG	NM_212779.1	901
	T7-*mpo*-R		TAATACGACTCACTATAGGGAGAAAGACGTTCGCAATGGTAGG		

Table. S2. Clinical manifestations of AML patients with high and low BM SOX4 expression

Variables	Total (n=112)	Low SOX4 Expression (n=62, 55.4%)	High SOX4 Expression (n=50, 44.6%)	*P* value
**Sex**†				0.4482
Male	63	37	26	
Female	49	25	24	
**Age (year)**‡	48	46	52	0.2277
**Lab data**‡				
WBC (/μL)	16945	15875	18740	0.4972
Hb (g/dL)	8.4	8.6	8.3	0.3111
Platelet (×1,000 /μL)	49.5	53	46.5	0.5046
Blast (/μL)	6011	4003	7787	0.4106
LDH (U/L)	955	881	1006	0.1025
**FAB**†				
M0	1	1	0	>0.9999
M1	30	18	12	0.6687
M2	34	20	14	0.6826
M3	14	8	6	>0.9999
M4/M5	29	13	16	0.2004
M6	4	2	2	>0.9999
**Karyotype***				
Favorable	29	20	9	0.0866
Intermediate	71	36	35	0.2321
Unfavorable	9	4	5	0.7286
**Genetic alteration**†				
NPM1	19	10	9	0.8055
FLT3/ITD	20	8	12	0.1437

†number of patients (%)

‡median

*109 patients, including 49 high SOX4 expression and 60 low SOX4 expression, had chromosome data at diagnosis.

Abbreviations: FAB, French-American-British classification; CR, complete remission; PR, partial remission

Statistically significant (P < 0.05)

Table. S3. Comparison of immune-phenotypes of leukemia cells between AML patients with high and low BM SOX4 expression

Antigens	Total patients examined	Percentage of patients with the antigen expression			*P* value
		Whole cohort	High SOX4 Expression	Low SOX4 Expression	
HLA-DR	111	73	73.5	73.7	>0.9999
CD13	110	97.3	95.8	98.4	0.5794
CD33	111	91.9	98	87.1	0.0749
CD14	111	7.2	10.2	4.8	0.2985
CD19	109	7.3	4.3	9.7	0.4619
CD11b	110	29.1	40.8	19.7	0.0202
CD7	109	16.5	10.4	21.3	0.1985
CD2	109	2.8	2.1	3.3	>0.9999
CD15	110	52.7	55.1	50.8	0.7034
CD34	111	68.5	71.4	66.1	0.8347
CD56	109	25.7	29.8	22.6	0.5072

Statistically significant (P < 0.05)

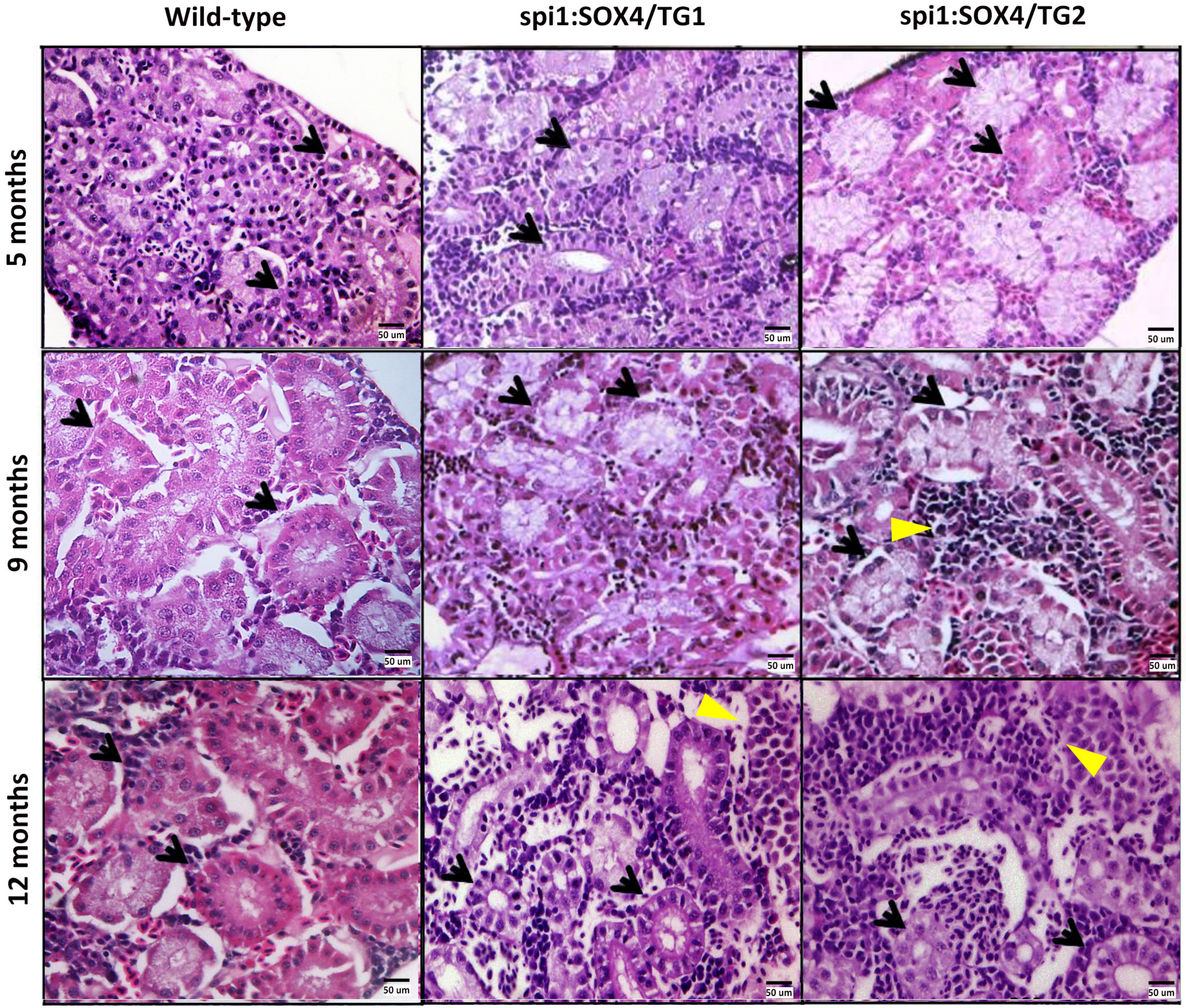
Wild-type
spi1:SOX4/TG1
spi1:SOX4/TG2
5 months
9 months
12 months
50 um

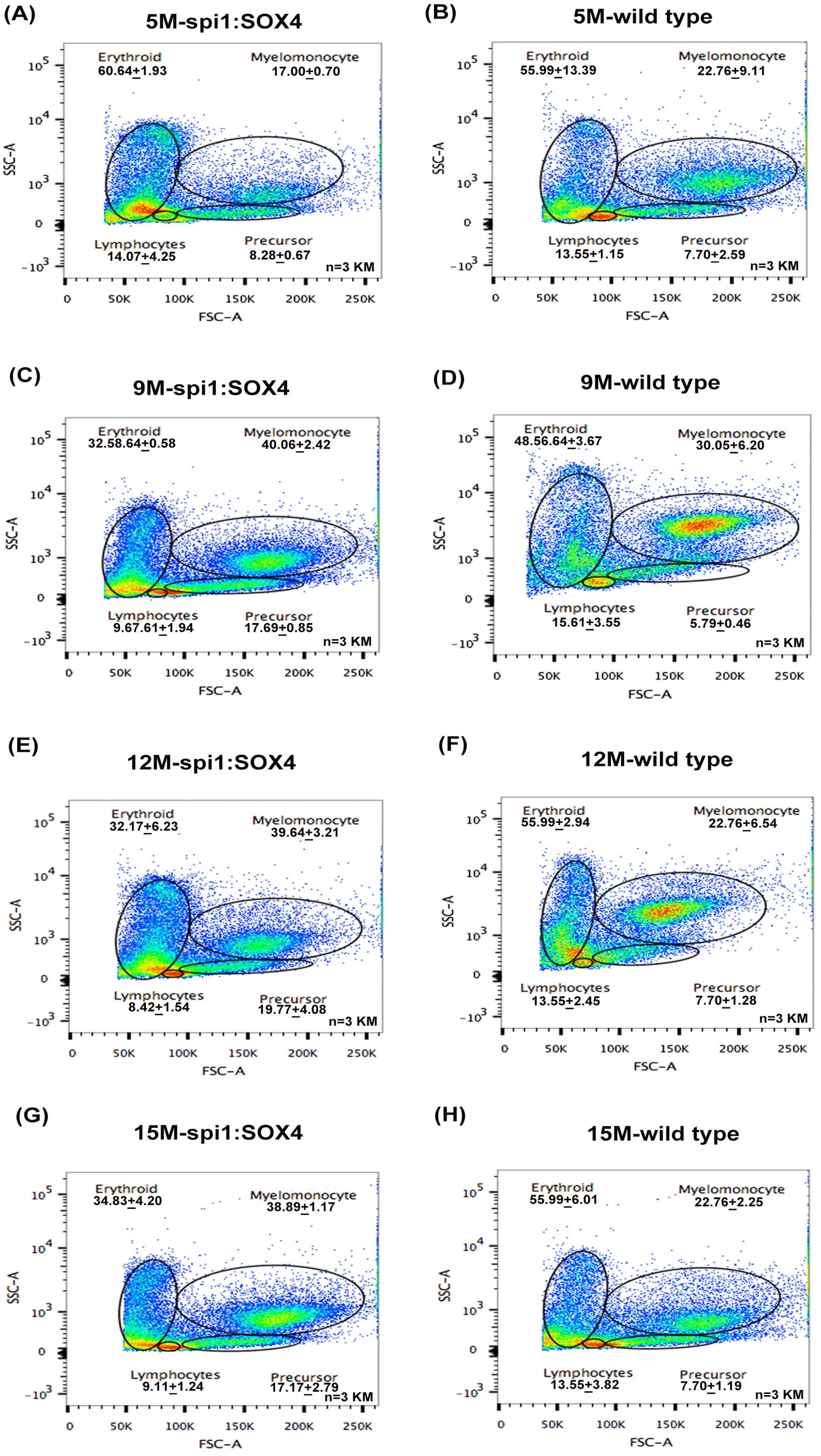

(A)
5M-spi1:SOX4
Erythroid
60.64±1.93
Myelomonocyte
17.00±0.70
Lymphocytes
14.07±4.25
Precursor
8.28±0.67
n=3 KM
SSC-A
FSC-A
(B)
5M-wild type
Erythroid
55.99±13.39
Myelomonocyte
22.76±9.11
Lymphocytes
13.55±1.15
Precursor
7.70±2.59
n=3 KM
SSC-A
FSC-A
(C)
9M-spi1:SOX4
Erythroid
32.58.64±0.58
Myelomonocyte
40.06±2.42
Lymphocytes
9.67.61±1.94
Precursor
17.69±0.85
n=3 KM
SSC-A
FSC-A
(D)
9M-wild type
Erythroid
48.56.64±3.67
Myelomonocyte
30.05±6.20
Lymphocytes
15.61±3.55
Precursor
5.79±0.46
n=3 KM
SSC-A
FSC-A
(E)
12M-spi1:SOX4
Erythroid
32.17±6.23
Myelomonocyte
39.64±3.21
Lymphocytes
8.42±1.54
Precursor
19.77±4.08
n=3 KM
SSC-A
FSC-A
(F)
12M-wild type
Erythroid
55.99±2.94
Myelomonocyte
22.76±6.54
Lymphocytes
13.55±2.45
Precursor
7.70±1.28
n=3 KM
SSC-A
FSC-A
(G)
15M-spi1:SOX4
Erythroid
34.83±4.20
Myelomonocyte
38.89±1.17
Lymphocytes
9.11±1.24
Precursor
17.17±2.79
n=3 KM
SSC-A
FSC-A
(H)
15M-wild type
Erythroid
55.99±6.01
Myelomonocyte
22.76±2.25
Lymphocytes
13.55±3.82
Precursor
7.70±1.19
n=3 KM
SSC-A
FSC-A

DATA POSTPROCESSING None
PROBE_NAME 201416_at [HG-U133A]
PROBE_DESCRIPTION 602579853F1 NIH_MGC_60 Homo sapiens cDNA clone IMAGE:4719060 5-, mRNA sequence
GENE_SYMBOL SOX4
GENE_DESCRIPTION SRY (sex determining region Y)-box 4

DATASET	GSE12417-GPL96
CANCER_TYPE	Blood cancer
SUBTYPE	AML
N	163
ENDPOINT	Overall Survival
PERIOD	Days
COHORT	AMLCG (1999-2003)
ARRAY TYPE	HG-U133A
CONTRIBUTOR	Metzeler
PRE-TREATMENT	Double-induction: 100%
CUTPOINT	0.78
MINIMUM *P*-VALUE	0.006012
CORRECTED *P*-VALUE	0.109329
$\ln(HR_{high} / HR_{low})$	0.60
COX *P*-VALUE	0.042882
ln(HR)	0.19
HR [95% CI]	1.21 [1.01 - 1.47]

EVENT

0	60	(36.8%)
1	103	(63.2%)

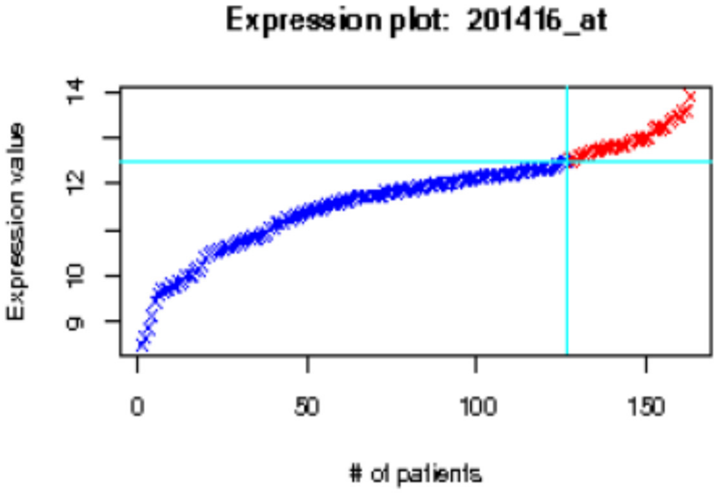

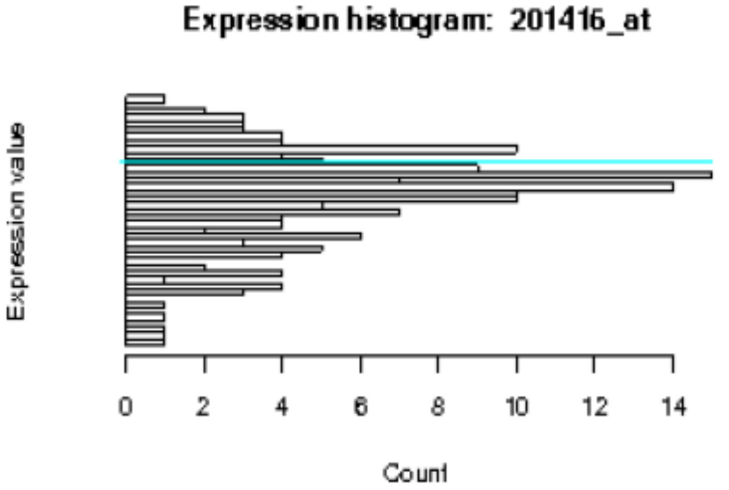

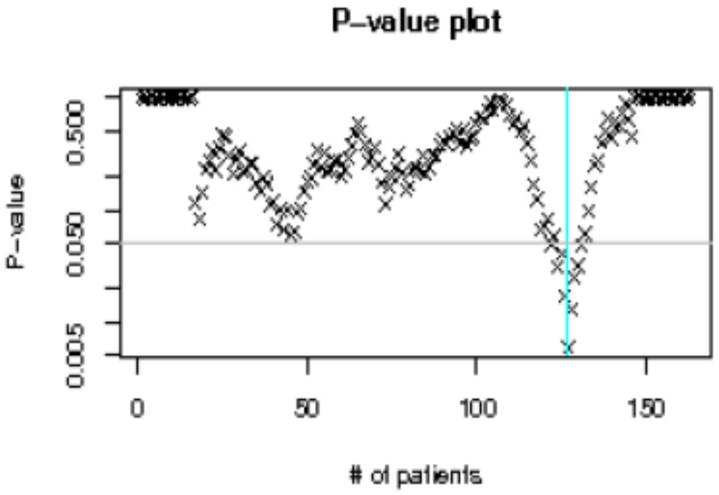

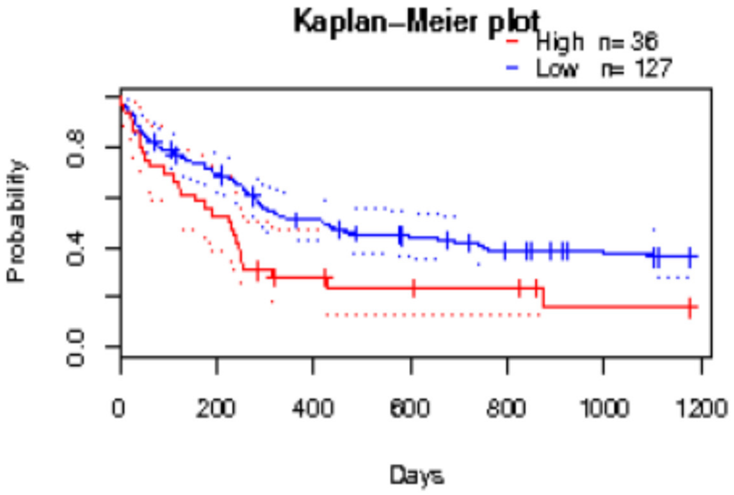

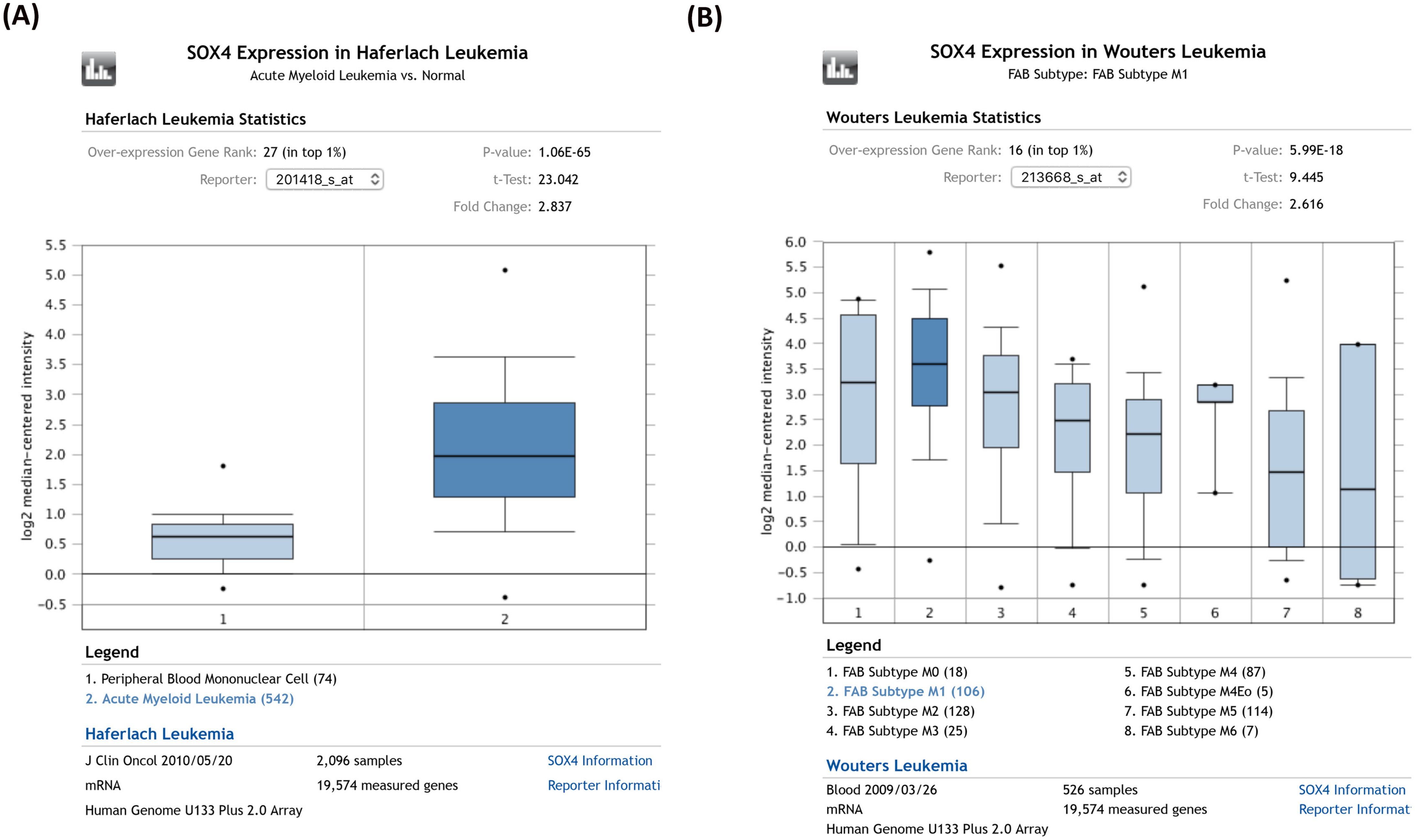
(A)
SOX4 Expression in Haferlach Leukemia
Acute Myeloid Leukemia vs. Normal
Haferlach Leukemia Statistics
Over-expression Gene Rank: 27 (in top 1%)
P-value: 1.06E-65
Reporter: 201418_s_at
t-Test: 23.042
Fold Change: 2.837
log2 median-centered intensity
5.5
5.0
4.5
4.0
3.5
3.0
2.5
2.0
1.5
1.0
0.5
0.0
−0.5
1
2
Legend
1. Peripheral Blood Mononuclear Cell (74)
2. Acute Myeloid Leukemia (542)
Haferlach Leukemia
J Clin Oncol 2010/05/20
2,096 samples
SOX4 Information
mRNA
19,574 measured genes
Reporter Informati
Human Genome U133 Plus 2.0 Array
(B)
SOX4 Expression in Wouters Leukemia
FAB Subtype: FAB Subtype M1
Wouters Leukemia Statistics
Over-expression Gene Rank: 16 (in top 1%)
P-value: 5.99E-18
Reporter: 213668_s_at
t-Test: 9.445
Fold Change: 2.616
log2 median-centered intensity
6.0
5.5
5.0
4.5
4.0
3.5
3.0
2.5
2.0
1.5
1.0
0.5
0.0
−0.5
−1.0
1
2
3
4
5
6
7
8
Legend
1. FAB Subtype M0 (18)
2. FAB Subtype M1 (106)
3. FAB Subtype M2 (128)
4. FAB Subtype M3 (25)
5. FAB Subtype M4 (87)
6. FAB Subtype M4Eo (5)
7. FAB Subtype M5 (114)
8. FAB Subtype M6 (7)
Wouters Leukemia
Blood 2009/03/26
526 samples
SOX4 Information
mRNA
19,574 measured genes
Reporter Informat
Human Genome U133 Plus 2.0 Array

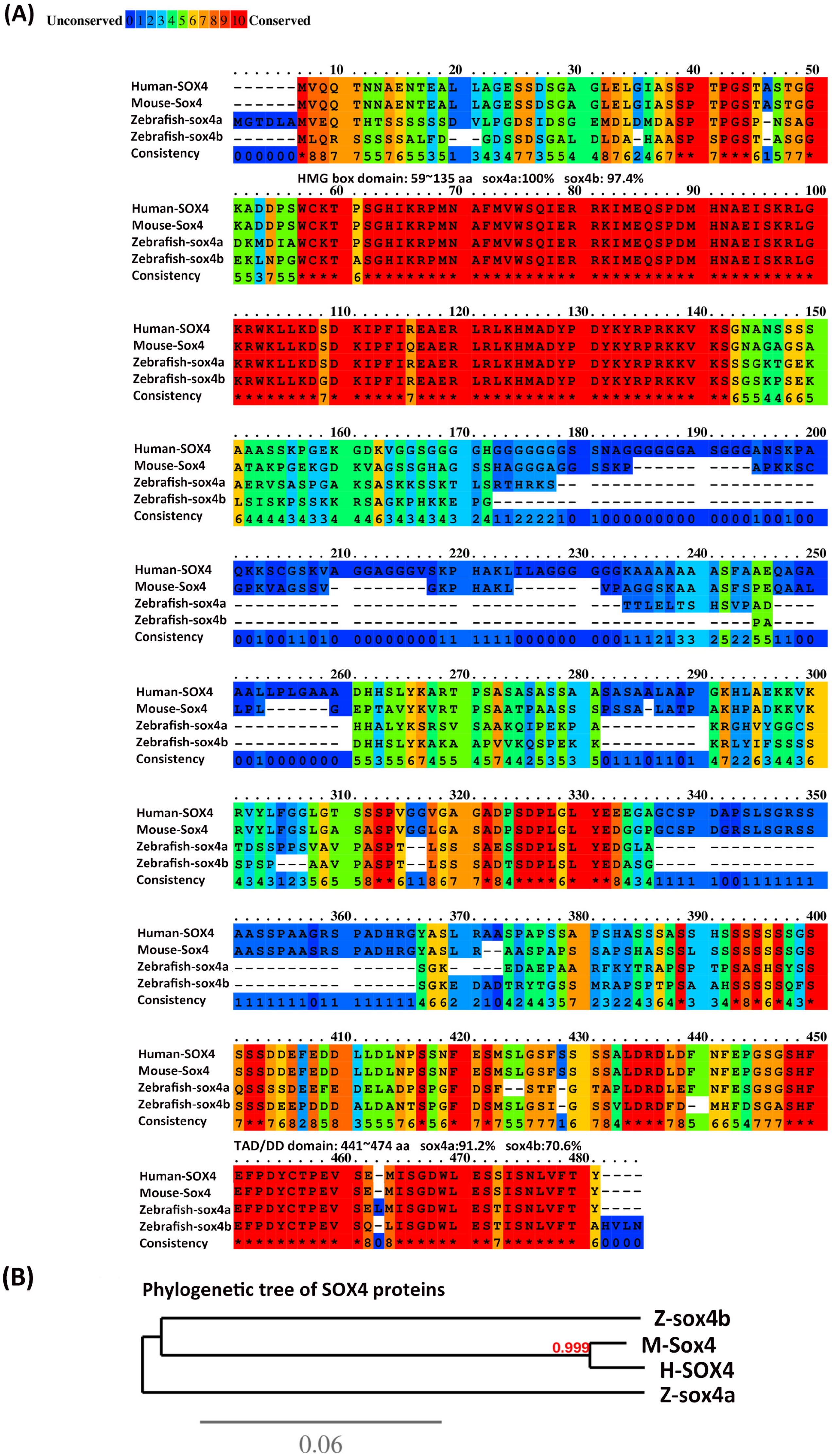

(A)
Unconserved 0 1 2 3 4 5 6 7 8 9 10 Conserved
Human-SOX4
Mouse-Sox4
Zebrafish-sox4a
Zebrafish-sox4b
Consistency
HMG box domain: 59~135 aa sox4a:100% sox4b: 97.4%
TAD/DD domain: 441~474 aa sox4a:91.2% sox4b:70.6%
(B)
Phylogenetic tree of SOX4 proteins
Z-sox4b
0.999
M-Sox4
H-SOX4
Z-sox4a
0.06
